# Loss of Melanopsin (OPN4) Leads to a Faster Cell Cycle Progression and Growth in Murine Melanocytes

**DOI:** 10.3390/cimb43030101

**Published:** 2021-10-04

**Authors:** Leonardo Vinícius Monteiro de Assis, Maria Nathália Moraes, Davi Mendes, Matheus Molina Silva, Carlos Frederico Martins Menck, Ana Maria de Lauro Castrucci

**Affiliations:** 1 Laboratory of Comparative Physiology of Pigmentation, Department of Physiology, Institute of Biosciences, University of São Paulo, São Paulo 05508-090, Brazil; nathalia.moraes@usp.br (M.N.M.); amdlcast@ib.usp.br (A.M.d.L.C.); 2 DNA Repair Lab, Department of Microbiology, Institute of Biomedical Sciences, University of São Paulo, São Paulo 05508-000, Brazil; davimendes4@gmail.com (D.M.); matheusmolina95@gmail.com (M.M.S.); cfmmenck@usp.br (C.F.M.M.); 3 Department of Biology, University of Virginia, Charlottesville, VA 22904, USA

**Keywords:** skin biology, melanocytes, opsins, melanopsin, proliferation and cell cycle, molecular clock

## Abstract

Skin melanocytes harbor a complex photosensitive system comprised of opsins, which were shown, in recent years, to display light- and thermo-independent functions. Based on this premise, we investigated whether melanopsin, OPN4, displays such a role in normal melanocytes. In this study, we found that murine *Opn4*^KO^ melanocytes displayed a faster proliferation rate compared to *Opn4*^WT^ melanocytes. Cell cycle population analysis demonstrated that OPN4^KO^ melanocytes exhibited a faster cell cycle progression with reduced G_0_–G_1_**,** and highly increased S and slightly increased G_2_/M cell populations compared to the *Opn4*^WT^ counterparts. Expression of specific cell cycle-related genes in *Opn4*^KO^ melanocytes exhibited alterations that corroborate a faster cell cycle progression. We also found significant modification in gene and protein expression levels of important regulators of melanocyte physiology. PER1 protein level was higher while BMAL1 and REV-ERBα decreased in *Opn4*^KO^ melanocytes compared to *Opn4*^WT^ cells. Interestingly, the gene expression of microphthalmia-associated transcription factor (MITF) was upregulated in *Opn4*^KO^ melanocytes, which is in line with a higher proliferative capability. Taken altogether, we demonstrated that OPN4 regulates cell proliferation, cell cycle, and affects the expression of several important factors of the melanocyte physiology; thus, arguing for a putative tumor suppression role in melanocytes.

## 1. Introduction

Melanocytes originate from neural crest cells during embryogenesis and are melanin-producing cells that inhabit the skin, but are also present in the inner ear, eyes, nervous system, and heart [1,2,3,4]. While the role of melanocytes in organs, other than the skin and eyes, are subject to debate [3], their function in the skin is widely comprehended. Melanocytes reside in the basal layer of the epidermis where each one interacts with 30 to 40 keratinocytes, creating the epidermal melanin unit (firstly described by [5]. In response to UV radiation and visible light, melanocytes synthesize the protective pigment melanin, which is transferred to neighboring keratinocytes where it acts as a physical shield against solar radiation [6,7,8]. Classically, in response to UV radiation and visible light, an increase in pigmentation takes place evoked after α-melanocyte stimulating hormone (α-MSH) interaction with melanocortin 1 receptor (MC1R) [9,10,11].

The microphthalmia-associated transcription factor (MITF) is a crucial player in melanocyte differentiation, development, survival, and proliferation as well as in the pigmentary responses. As a transcription factor, MITF regulates specific gene programs that lead to development and differentiation; thus, affecting a myriad of biological processes in melanocytes [12,13]. In addition to exerting an important physiological role, MITF is also an important player in melanoma biology as it is didactically explained by a rheostat model: high, intermediate, and low levels of MITF lead to differentiated, proliferative, and invasive phenotypes, respectively, whereas MITF absence results in senescence or cell death [14,15,16,17].

An important feature of melanocytes is their sensitivity to UV and light stimulus responding with important physiological processes, mainly pigmentation. Most of the literature has focused on analyzing the endpoint of such response, i.e., pigmentation, proliferation, DNA damage, and others, while just a handful of studies have evaluated how melanocytes are actually able to sense light and UV radiation photons. Within this line of thought, opsins—light sensing molecules—known to be expressed in the eye, where they participate in visual and non-visual processes [18,19,20,21,22], were first demonstrated in the skin in early 2000 in mice [23] and 2009 in humans [24]. Functional studies were only performed almost a decade later by Oancea’s lab pioneering reports [25,26,27]. To the present day, the photosensitive system of the skin has been shown to participate in murine and human: pigmentary responses [25,26,27,28,29,30,31], differentiation processes of keratinocytes [32,33], hair follicle growth [34], UVA-induced photoaging [35], cellular growth and apoptosis in response to UVA radiation [28], and UV- and blue light-induced calcium influx [25,27,36].

In recent years, the paradigm of opsins being light sensors was challenged by studies in murine melanocytes demonstrating that melanopsin can also detect thermal energy [37]. In addition, it was shown that sperm cell thermotaxis is dependent on OPN2 and OPN4 presence [38,39]. More recently, light- and thermo-independent roles of opsins have also been reported in human melanocytes, thus, revealing an even more complex scenario for opsin signaling. For instance, OPN3 has been associated with negative regulation of the MC1R pathway, leading to an inhibitory effect on melanogenesis [40] as well as *Opn3* knockdown resulted in melanocyte apoptosis [41]. OPN5 has also been implicated as a negative regulator of melanogenesis since its downregulation by gene silencing resulted in reduced expression of key enzymes involved in melanin synthesis in a UV-independent manner [42]. In this study, we demonstrate a light- and thermo-independent role of OPN4 in murine melanocytes harboring a functional (*Opn4*^WT^) and non-functional (*Opn4*^KO^) OPN4 protein by evaluating cellular metabolism, proliferation, and cell cycle regulation.

## 2. Material & Methods

### 2.1. Cell Culture

*Opn4*^KO^ Melan-a melanocytes were generated using Clustered Regularly Interspaced Short Palindromic Repeats (CRISPR) technique. Cells underwent phenotypic characterization and Sanger sequencing revealed a disruption of one *Opn4* allele that rendered these cells OPN4 impaired, as previously described in detail [28]. *Opn4*^WT^ and *Opn4*^KO^ cells were subject to *Per1*: *Luc* gene transfection as described previously [28] and were also used in this study.

Cells were cultured in RPMI 1640 medium without phenol red (Atena, Brazil), supplemented with 25 mM NaHCO_3_ (Sigma-Aldrich, St. Louis, MO, USA), 20 mM HEPES (Santa Cruz, Dallas, TX, USA), 10% fetal bovine serum (FBS, Atena, Campinas, Sāo Paulo, Brazil), 1% antibiotic/antimycotic solution (10,000 U/mL penicillin, 10,000 μg/mL streptomycin, and 25 μg/mL amphotericin B, Thermo Fisher, Waltham, MA, USA), at pH 7.2. Cells were kept at 37 °C with 5% CO_2_ Geneticin (G418, Thermo Fisher, Waltham, MA, USA) was used to guarantee selection only during maintenance. In experiments, cells were kept in the same medium with reduced (2%) FBS, and when kept in the absence of CO_2_, HEPES was increased to 50 mM. In maintenance and experimental conditions, phorbol 12- myristate 13-acetate (PMA, 200 nM) and *all-trans* retinal (100 nM) were added (both from Sigma-Aldrich, St. Louis, MO, USA). Cell manipulation during the experiments was carried in the dark under red dim light (7W Konex bulb and Safelight filter GBX-2, Kodak, Rochester, NY, USA).

### 2.2. XTT Metabolic Assay

For XTT experiments, 2 × 10^4^ *Opn4*^WT^ or *Opn4*^KO^ melanocytes were seeded per well in 96-well plates in 100 µL of experimental medium and kept for 24 h at 37 °C with 5% CO_2_. On the following day, 50 µL of XTT and electron coupling reagent (ECR) solution (7:1) were immediately added. Cells were kept at 37 °C with 5% CO_2_ for another 24 h, and then the absorbance was read in a spectrophotometer (SpectraMax 250, Molecular Devices, San José, CA, USA). Specific absorbance was calculated as (A_450 sample -_ A_450 blank_) - A_660_ according to the manufacturer’s instructions (CyQUANT™ XTT Cell Viability Assay, Thermo Fisher, Waltham, MA, USA).

### 2.3. Cellular Growth Assay

One thousand *Opn4*^WT^ or *Opn4*^KO^ melanocytes were seeded per well in 12-well plates in 2 mL of experimental medium and kept for 24 h at 37 °C with 5% CO_2_; 24, 48, 72, and 96 h later, the cells were harvested with Tyrode/EDTA solution and counted in a hemocytometer.

For gene expression, melanin quantification, and flow cytometry experiments, the same experimental setup was made and the cells were harvested with Tyrode/EDTA solution 4 days after seeding and processed as described below. Doubling time was calculated using the online tool available at https://www.doubling-time.com/compute.php (accessed on 22 June 2020). 

### 2.4. Melanin Content Assessment

At the end of the experiment, a 500 µL aliquot of the medium was collected in a tube containing 500 µL of 1M NaOH (in 10% DMSO), and heated at 80 °C for 60 min. Then, tubes were centrifuged at 1050× *g* for 15 min, and the supernatant was collected. For intracellular melanin, cells were harvested using Tyrode/EDTA solution and centrifuged at 100× *g* for 5 min. NaOH was added to the cell pellet, heated at 80 °C for 60 min, then centrifuged at 1050× *g* for 15 min, and the supernatant was collected. Each sample was added in duplicate to wells of a 96-well flat-bottom plate, and total absorbance was measured at 475 nm. The values were interpolated in a standard curve of synthetic melanin (Sigma-Aldrich, St. Louis, MO, USA), ranging from 1.5625 μg/mL to 40 μg/mL. Melanin content was expressed as the sum of intra- and extracellular melanin divided by cell number.

### 2.5. Flow Cytometry

For cell cycle evaluation, cell staining was based on the manufacturer’s instructions (BD Biosciences, Franklin Lakes, NJ, USA). In brief, cells were loaded with BrdU (an S phase marker) solution (10 mM) for 2 h and kept at 37 °C with 5% CO_2_. Cells were harvested using Tyrode/EDTA solution, fixed using Cytoperm Cytofix solution (BD Biosciences, Franklin Lakes, NJ, USA) on ice for 30 min, and washed with Perm/Wash buffer. Then, approximately 10^5^–5 × 10^5^ cells were placed into each well of a 96-well round bottom plate, fixed again with Cytoperm Permeabilization Buffer Plus on ice for 10 min, and washed with Perm/Wash buffer. Cytofix/Cytoperm Buffer was again applied to the cells on ice for 5 min and cells were washed. Then, DNase (300 µg/mL) was added, cells were placed at 37 °C for 1 h, washed, resuspended in anti-BrdU antibody in Perm/Wash buffer (FITC, 1:50), and kept at room temperature for 20 min. Cells were then washed, resuspended in 7-AAD solution (for DNA staining), and kept in staining buffer until the acquisition in Canto Flow Cytometry apparatus (BD Biosciences, Franklin Lakes, NJ, USA).

For protein evaluation, cells were harvested with Tyrode/EDTA solution and fixed with Cytoperm Cytofix solution (BD Biosciences, Franklin Lakes, NJ, USA) on ice for 30 min. Cells were washed with Perm/Wash buffer (BD Biosciences, Franklin Lakes, NJ, USA), approximately 10^5^–5 × 10^5^ cells were added per well in 96-well round bottom plates and blocked with PBS containing 1% of bovine serum albumin (BSA) at room temperature for 30 min. Cells were washed and incubated overnight in PER1 (ABCAM, USA, ab136451, 1:200), BMAL1 (ABCAM, ab93806, 1:200), or REV-ERBα (Novus Biological, Minneapolis, Minnesota, USA, NBP2-19574, 1:200) antibodies in Perm/Wash buffer. On the next day, cells were washed, and a secondary anti-rabbit antibody (Alexa Fluor 488, Thermo Fisher, Waltham, MA, USA) was added at room temperature for 60 min. Cells were washed and resuspended in staining buffer, kept at 4 °C, and then read in a Canto Flow Cytometry (BD Biosciences, Franklin Lakes, NJ, USA). For BMAL1 and REV-ERBα staining, 0.5% Triton X-100 was added to allow nuclear permeabilization, which was not required for PER1 staining. At least 10^4^ events were captured, cell doublets were excluded by analyzing FSC-H versus FSC-A. Non-stained controls were used to exclude cellular autofluorescence. Data was analyzed in FlowJO software (BD Biosciences, Franklin Lakes, NJ, USA). Percentage of positive cells and median intensity fluorescence (MIF) were exported and analyzed with PRISMA 7.0 (GraphPad, San Diego, CA, USA).

### 2.6. RNA Extraction and CDNA Synthesis

The medium was removed and TRIzol (Thermo Fisher, Waltham, MA, USA) was added onto the cells, collected, and stored at −80 °C until processing. RNA was extracted using 1-bromo-3-chloropropane (Sigma, St. Louis, MO, USA), precipitated with isopropanol (Sigma, St. Louis, MO, USA), and washed with 75% molecular grade ethanol (Sigma, St. Louis, MO, USA). RNA pellets were resuspended in DEPC water and genomic contamination was prevented using *TURBO DNase* (Thermo Fisher, Waltham, MA, USA). RNA concentration and quality (OD_260_/OD_280_) were assessed in a spectrophotometer (NanoDrop, Wilmington, DE, USA). One μg of total RNA was subject to reverse transcriptase reaction using random primers and Superscript III, in addition to the reagents recommended by the enzyme manufacturer (Thermo Fisher, Waltham, MA, USA).

### 2.7. Quantitative PCR (qPCR)

Twenty-five ng of cDNA was subject to quantitative PCR using species-specific primers (Table 1) spanning introns, based on sequences obtained from GenBank (http://www.ncbi.nlm.nih.gov/genbank (accessed on 23 May 2020)), designed by Primer Blast (http://www.ncbi.nlm.nih.gov/genbank (accessed on 23 May 2020)) or Primer Quest (IDT, Coralville, IA, USA), and synthesized by Integrated DNA Technologies (IDT, Coralville, IA, USA). *Rpl37a* was used to normalize the expression values of the genes of interest.

For simultaneous analysis of *Per1* and *Bmal1* or *Opn2* and *Clock*, multiplex reactions containing cDNA, primers, fluorescent probes, and Kapa Probe Fast Mix (Kapa Biosystems, USA) were used and run in triplicates for each experimental cDNA sample. Reactions were carried out in an iQ5 thermocycler (Bio-Rad Laboratories, Hercules, CA, USA) in the following conditions: 3 min at 95 °C followed by 45 cycles of 15 s at 95 °C and 60 s at 60 °C. For the other genes, independent solutions were prepared with cDNA (25 ng), specific primers, and Kapa Sybr Fast Mix, and run in duplicates in an iQ5 thermocycler. Reactions were subject to the following conditions: 10 min at 95 °C, followed by 45 cycles of 15 s at 95 °C, 60 s at 60 °C, and 80 cycles of 10 s at 55 °C with a gradual increase of 0.5 °C.

### 2.8. Bioluminescence Experiments

One hundred thousand *Per1: Luc Opn4*^WT^ or *Opn4*^KO^ melanocytes were seeded in 35 mm dishes in the experimental medium and kept at 37 °C with 5% CO_2_ for 24 h. On the next day, cells were treated with 200 nM dexamethasone or 10 µM FSK, 100 µM luciferin (Promega, Madison, WI, USA) was added, dishes were sealed with 35 mm round coverslips and parafilm (VWR, Radnor, PA, USA), and placed in the Lumicycle equipment (Actimetrics, Wilmette, IL, USA) in an incubator without CO_2_ positive pressure at 37 °C. Drugs remained in the medium until the end of the experiment. Bioluminescence was recorded and the temperature of the incubator was monitored (iLog, Escort Data Loggers, Auckland, New Zeeland) every 10 min.

### 2.9. Statistical Analysis

XTT data, gene and protein expression, as well as cell cycle results, were analyzed by unpaired Student’s *t*-test. Cellular growth was analyzed by two-way ANOVA followed by Bonferroni post-test. For bioluminescence analysis, the amplitude was calculated by subtracting the lowest values from the highest values within the first 24 h and analyzed by unpaired Student’s *t*-test. Data obtained from at least two independent experiments (N) were used for all statistical analyses. The number of samples (n) is shown in each figure legend. *p* values < 0.05 were used to reject the null hypothesis and were calculated in GraphPad Prism 7.0.

## 3. Results

### 3.1. Cellular Proliferation Is Higher in the Absence of Opn4

We initially evaluated the impact of *Opn4* deletion in normal melanocytes, which were gene-edited by the Clustered Regularly Interspaced Short Palindromic Repeats (CRISPR) technique and further validated by Sanger sequencing, as previously reported [28]. Among three independent clones, all showing functional evidence of OPN4 impairment, one clone was selected and used in this study.

**Figure 1:**
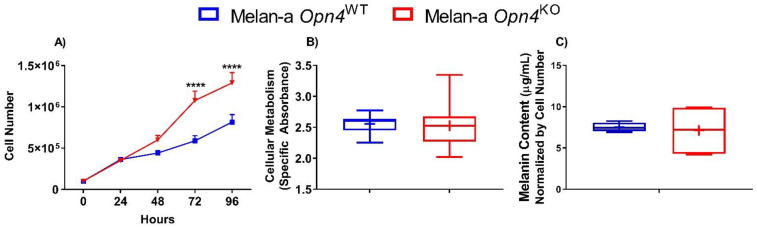
Cellular proliferation, metabolism, and melanin content of *Opn4*^WT^ and *Opn4*^KO^ normal melanocytes. (**A**) Cellular proliferation along 4 days (*n* = 9); (**B**) specific absorbance of XTT after 24 h (*n* = 10–21); (**C**) total (intra- and extra-cellular) melanin content on the fourth day (*n* = 4–5). **** *p* < 0.0001.

The initial step was to determine the metabolic activity, cellular growth, and melanin content of *Opn4*^WT^ and *Opn4*^KO^ melanocytes. We found that *Opn4*^KO^ melanocytes displayed a higher cell number compared to *Opn4*^WT^ cells after 3 days in culture, a difference maintained in the following day (Figure 1A). Indeed, the proliferation rate of both genotypes is significantly different as *Opn4*^KO^ melanocytes showed a faster doubling time (~34 h) compared to *Opn4^WT^* normal melanocytes (~47 h). On the other hand, mitochondrial metabolism of XTT, commonly used as a cellular proxy of proliferation and metabolism, did not show any difference between *Opn4*^WT^ and *Opn4*^KO^ melanocytes (Figure 1B). As to the melanin-producing capacity in the absence or presence of OPN4, no difference between *Opn4*^WT^ and *Opn4*^KO^ melanocytes was found. (Figure 1C), which was previously shown under different experimental conditions [28].

### 3.2. Opn4^KO^ Melanocytes Display a Faster Cell Cycle Progression with Important Alterations in Cell Cycle-Related Genes

**Figure 2:**
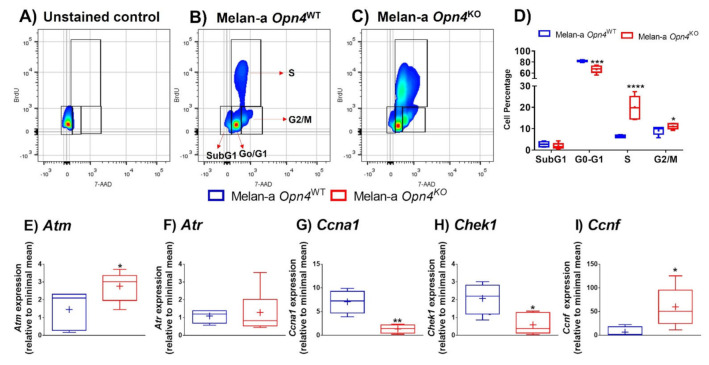
Cell cycle evaluation by 7-AAD and BrdU staining and by the expression of cell cycle-related genes in *Opn4*^WT^ and *Opn4*^KO^ melanocytes. (**A**–**C**) Representative gate of 7-AAD and BrdU stained cells; (**D**) quantitative analysis of cell cycle phases (*n* = 5–6); (**E**–**I**) gene expression (*n* = 4–8). * *p* < 0.05; ** *p* < 0.01; *** *p* < 0.001; **** *p* < 0.0001.

Our next step was to evaluate the cell cycle phases in *Opn4*^WT^ and *Opn4*^KO^ melanocytes using 7-AAD and BrdU. The results clearly demonstrated that *Opn4*^KO^ melanocytes have a reduced (18%) cell population in the G_0_-G_1_ phase, but massively increased cell number in the S phase (300%), and a slightly increased (20%) in the G_2_/M phase compared to *Opn4*^WT^ melanocytes (Figure 2A–D). These findings associated with the proliferation data (Figure 1A) suggest that cell cycle progression is accelerated in the absence of *Opn4*.

Based on these data, we analyzed the expression of some key cell cycle-related genes in *Opn4*^WT^ and *Opn4*^KO^ melanocytes: ataxia-telangiectasia-mutated (*Atm*) and ataxia telangiectasia and Rad3-related (*Atr*), which encode proteins that act on DNA damage response and are responsible for maintaining genome integrity [43]. Upon ATM and ATR activation, both proteins lead to increased expression of cell cycle checkpoint pathways that may result in cell cycle arrest and DNA repair. ATM and ATR primarily respond to double- and single-strand DNA breaks, respectively [43]. In our cell model, *Atm* expression was upregulated, while *Atr* was not affected in *Opn4*^KO^ melanocytes compared to *Opn4*^WT^ cells (Figure 2E,F).

The protein encoded by *Ccna1* (Cyclin 1) is a member of the cyclins, which are known to be important regulators of the cell cycle due to their ability to bind and activate cyclin-dependent kinases (CDKs). It has been reported that Cyclin 1 expression increases during cell cycle progression reaching its highest levels in S and G_2_/M phases [44,45]. Despite its positive role in cell cycle progression, we found a reduction of *Ccna1* expression in *Opn4*^KO^ melanocytes compared to *Opn4*^WT^ counterparts (Figure 2G). Since *Opn4*^KO^ melanocytes showed a large increase in the S phase population, one might suggest that the decreased *Ccna1* expression may be the result of a compensatory effect. *Checkpoint kinase 1* (*Chek1*) gene encodes a serine/threonine-specific protein kinase that participates in cell cycle events in response to DNA damage, mainly leading to cell cycle arrest, DNA repair, and cellular death [46]. In an undisturbed cell cycle, CHEK1 is also an important regulator of G_2_/M progression, and is activated by cyclin B. CHEK1 is known to regulate replication checkpoint in the G_2_/M phase and is required for S phase progression and cell survival [46]. In our model, a reduction of *Chek1* expression was found in *Opn4*^KO^ melanocytes in comparison to *Opn4*^WT^ cells (Figure 2H), which corroborates our data of a faster cell cycle progression in the absence of *Opn4*. Cyclin F, encoded by *Ccnf,* plays an important role as an activator of cell cycle progression [47,48]. In our experimental model, *Opn4*^KO^ melanocytes displayed increased expression of *Ccnf* when compared to *Opn4*^WT^ cells (Figure 2I), which is in line with a faster cell cycle progression displayed by *Opn4*^KO^ melanocytes.

Collectively, we show evidence that *Opn4* participates as a cell cycle regulator since a faster progression, seen by decreased G_0_/G_1_, increased S and G_2_/M cell populations, was demonstrated in *Opn4*^KO^ cells. Importantly, in line with the cell cycle data, gene expression of *Chek1*, an important S and G_2_/M regulator, and *Ccnf*, a cell cycle activator, are down and upregulated, respectively, in *Opn4*^KO^ melanocytes compared to *Opn4*^WT^ ones.

### 3.3. Molecular Clock Activation Is Impaired in the Absence of Opn4

**Figure 3:**
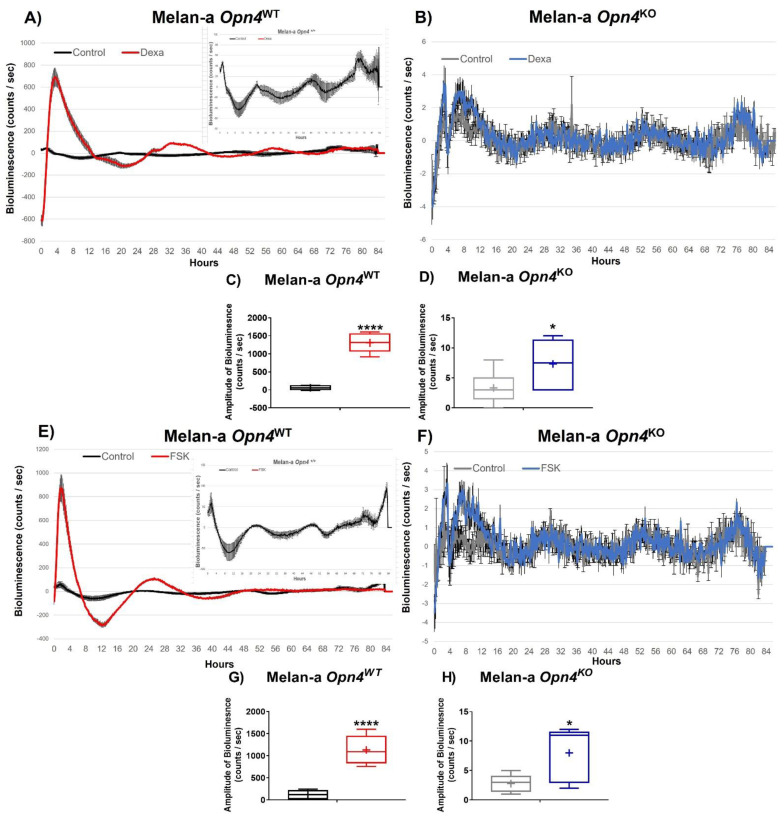
*Per1*:Luc bioluminescence of *Opn4*^WT^ and *Opn4*^KO^ melanocytes treated with dexamethasone (**A**–**D**) or forskolin (**E**–**H**). (**A**,**B**,**E**,**F**) Representative graphs of bioluminescence. Inserts represent the untreated control groups in a different scale; (**C**,**D**,**G**,**H**) amplitude of bioluminescence. (*n* = 5–6). * *p* < 0.05; **** *p* < 0.0001.

**Figure 4:**
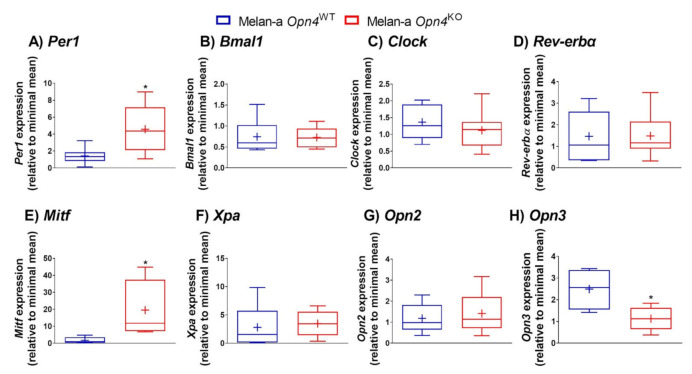
Gene expression of clock genes (**A**–**D**), *Mitf* (**E**), *Xpa* (**F**), *Opn2* (**G**), and *Opn3* (**H**) in *Opn4*^WT^ and *Opn4*^KO^ melanocytes. (*n* = 4–8). * *p* < 0.05.

As in the absence of *Opn4*, an increase in cellular proliferation was found; we investigated the participation of the molecular clock in this response since clock genes play an important regulatory role in melanocytes [49]. We first used dexamethasone, a synthetic glucocorticoid receptor agonist, widely recognized for its ability to activate the molecular clock [50]. Upon dexamethasone treatment, *Opn4*^WT^ melanocyte *Per1* bioluminescence acutely increased, displaying almost 15-fold the bioluminescence of the untreated control *Opn4*^WT^ melanocytes (Figure 3A,C). On the other hand, *Opn4*^KO^ melanocytes exhibited a marked suppression of *Per1*-induced dexamethasone effects, displaying a slight increase of the bioluminescence amplitude compared to the untreated control (Figure 3B,D). Similar findings were found with another classic molecular clock activator, forskolin (FSK) [50]. FSK treated *Opn4*^WT^ melanocytes acutely and significantly increased *Per1* bioluminescence compared to the untreated control (Figure 4E,G). In *Opn4*^KO^ melanocytes, FSK led to a slight increase of *Per1* bioluminescence compared to the control (Figure 4F,H). Of note, the absence of marked rhythms in the above-described groups may be due to the maintenance of the drugs in the medium throughout the experiment.

Taken altogether, these data show that dexamethasone and FSK can activate the molecular clock; however, such activation is less pronounced in the absence of OPN4.

### 3.4. Expression of Molecular Clock Components, Microphthalmia-Associated Transcription Factor (Mitf), and Panopsin (Opn3) Is Altered in the Absence of Opn4

**Figure 5:**
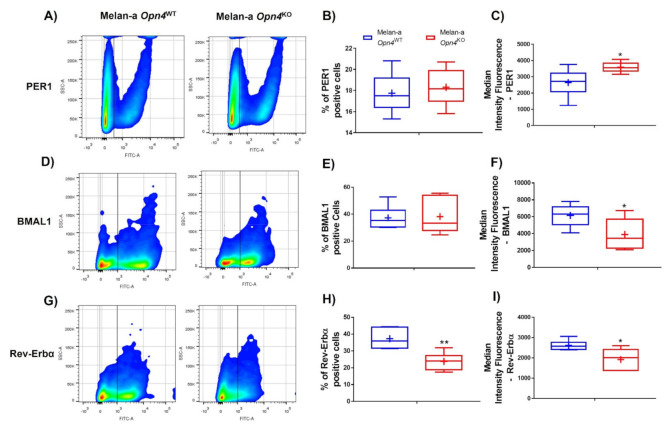
PER1, BMAL1, and REV-ERBα protein evaluation using specific antibodies in flow cytometry in *Opn4*^WT^ and *Opn4*^KO^ melanocytes. (**A**,**D**,**G**) Representative gates of PER1-, BMAL1-, and REV-ERBα-stained cells; (**B**,**E**,**H**) percentage of positive cells for a given protein; (**C**,**F**,**I**) median intensity fluorescence (MIF). (*n* = 4–6). * *p* < 0.05. ** *p* < 0.01.

The next step was to assess gene and/or protein expression of some important components of the molecular clock known to play an important regulatory role in skin cells and melanocytes [28,29,37,51,52,53]. *Opn4*^KO^ melanocytes showed an increase of *Per1* mRNA expression compared to *Opn4*^WT^ melanocytes (Figure 4A). Interestingly, flow cytometry showed no alteration in the frequency of PER1 positive cells (Figure 5A,B), but an increase of protein fluorescence was detected in *Opn4*^KO^ melanocytes compared to *Opn4*^WT^ cells (Figure 5A,C). The mRNA expression of other clock genes such as *Bmal1*, *Clock*, and *Rev-erbα* did not show any difference between the genotypes (Figure 4B–D), although a reduction of BMAL1 protein level was detected (Figure 5D,F), with no alteration on the frequency of BMAL1 positive cells (Figure 5E), in *Opn4*^KO^ melanocytes compared to wild type cells. On the other hand, the frequency and fluorescence of REV-ERBα protein-positive cells in the *Opn4*^KO^ melanocyte population were reduced compared to *Opn4*^WT^ melanocytes (Figure 5G–I).

As we observed marked differences in cellular proliferation, we evaluated the expression of *Mitf* that plays a master regulatory role in melanogenesis, cell cycle, survival, metabolism, and differentiation of melanocytes [12]. Interestingly, *Mitf* mRNA expression was upregulated by almost 12-fold in *Opn4*^KO^ compared to *Opn4*^WT^ cells (Figure 4E). Xeroderma Pigmentosum, Complementation Group A gene, *Xpa*, expression has been shown to display a rhythmic expression pattern in mouse skin and melanocytes [54,55], which results in higher UVB carcinogenic effects in the morning compared to the evening [54]. In our study, *Xpa* mRNA expression was not different between the genotypes (Figure 4F).

We have previously demonstrated that UVA-induced pigmentary response in melanocytes is dependent on a cooperative action between OPN2 and OPN4 [30]. Thus, we questioned whether a putative compensatory mechanism would impact *Opn2* as well as panopsin (*Opn3*) in the absence of functional OPN4. We did not detect any difference in *Opn2* mRNA expression between *Opn4*^WT^ and *Opn4*^KO^ cells (Figure 4G), however, *Opn3* mRNA expression was clearly reduced in *Opn4*^KO^ compared to the wild type melanocytes (Figure 4H). OPN3 is a widely expressed opsin with roles in apoptosis and autophagy [56,57] and negative regulation of melanogenesis [40]. Therefore, the absence of *Opn4* leads to important alterations in the expression of molecular clock genes, *Mitf* and *Opn3* genes, which strengthens an unexpected regulatory role of *Opn4* in a light- and thermo-independent fashion.

## 4. Discussion

Opsins have been classically associated with light-sensing ability and their role in visual and non-visual biological processes [18,19,20,21,22]. In particular, the skin is an interesting peripheral organ in which opsin expression was first reported almost 20 years ago [23]. Since then, an increasing number of studies have demonstrated the presence and functionality of opsins in the skin [24,25,26,27,28,29,30,31,33,34,35,36,40,41,42,52,58]. Recently, the role of opsins in the skin and other peripheral tissues has been reviewed [59,60].

In addition to being a light sensor, groundbreaking data in *Drosophila* first showed evidence that opsins may also act as thermosensors [61,62]. Such a concept was also shown to take place in mammals: sperm cell thermotaxis has been demonstrated to be dependent on OPN2 and OPN4 [38,39]. Our group has also demonstrated that OPN4 detects high temperature in normal and malignant cutaneous melanocytes, leading to molecular clock activation [37]. Therefore, the above-mentioned studies provided an important paradigm change, i.e., opsins are not only light sensors.

Recently, a more complex scenario has been drawn as opsins in *Drosophila* were shown to act as chemosensors in a light- and thermo-independent fashion [63]. Within this line of thought, light- and thermo-independent functions of opsins have been demonstrated by some studies in mammalian cells. In human melanocytes, OPN3 interacts with the melanocortin 1 receptor, leading to a negative effect on melanogenesis in a light-independent manner [40]. In addition, *OPN3* silencing in human melanocytes evokes cellular apoptosis by modulating several pro- and anti-apoptotic proteins [41]. To the best of our knowledge, no study has addressed the putative light- and thermo-independent roles of OPN4.

The data provided in this study show that the absence of *Opn4* results in increased cellular growth, despite no difference in metabolic activity, compared to *Opn4*^WT^ cells. Interestingly, dexamethasone led to an acute increase of *Per1* bioluminescence with molecular clock activation in *Opn4*^WT^ cells, which is much higher than in *Opn4*^KO^ cells. A similar scenario was also found for FSK, i.e., marked suppression of *Per1* bioluminescence in *Opn4*^KO^ cells compared to *Opn4*^WT^. Although, the absence of *Opn4* may have led to the suppression of dexamethasone and FSK effects, it is possible that this difference will be due to experimental limitations such as different degrees of luciferase gene expression by each cell type.

In terms of phenotype, the absence of OPN4 significantly affected the cell cycle as *Opn4*^KO^ melanocytes displayed a faster cell cycle progression with reduced G_0_–G_1_, and highly increased S-phase and slightly increased G_2_/M- phase cell populations. Upon evaluation of selected cell cycle-related genes, our data suggest that *Opn4*^KO^ melanocytes display an altered expression pattern of cell cycle factors that contribute to the faster cell cycle progression found. Indeed, the reduced *Chek1*, an important S and G_2_/M regulator, and increased *CcnF*, a known cell cycle activator, expression ultimately results in a faster cell cycle progression. Another interesting finding is the reduced expression of *Opn3* in *Opn4*^KO^ melanocytes since *Opn3* gene silencing has been shown to negatively affect melanogenesis [40] and to lead to apoptosis [41]. In fact, altered pigmentation and cellular death were not found in our previous report in *Opn4*^WT^ and *Opn4*^KO^ cells [28]. In that study, the subG1 population was not affected by *Opn4* absence either; thus, ruling out that *Opn4* may act similarly as *Opn3* in this experimental model.

We also investigated how the lack of *Opn4* affected important genes related to the molecular clock, proliferation, pigmentation, DNA repair, and other opsins. Interestingly, we found that the absence of a functional *Opn4* increased gene and protein expression of PER1 and reduced BMAL1 and REV-ERBα protein levels. It is a known fact that decrease or increase of gene/protein expression of the clock molecular components results in severe biological outcomes, which are dependent but not limited to, cell phenotype, healthy or cancerous cell line, experimental conditions etc., [64,65,66]. Our data show how the lack of functional *Opn4* affects the molecular clock components and their responsiveness to classical clock activators.

Remarkably, *Mitf* expression was highly upregulated in *Opn4*^KO^ melanocytes compared to *Opn4*^WT^ cells. The global phenotype displayed by *Opn4*^KO^ melanocytes may reside in increased *Mitf* as MITF is an important transcription factor that ultimately regulates differentiation, cell cycle, pigmentation, survival, and metabolism of melanocytes [12]. MITF role in melanoma can be seen as a rheostat in which high levels lead to a differentiated phenotype whereas intermediate and low levels result in proliferative and invasive phenotypes [14,15,16,17]. In melanocytes, MITF alteration seems to be an early event [67] linked to serine/threonine-protein kinase B-Raf (BRAF) activation [68,69], which is also an early event in melanoma genesis [68,69]. The absence of *Opn4* resulted in *Mitf* increased gene expression, thus affecting several pathways, culminating in a higher proliferative capacity; however, melanin content was not affected by the absence of *Opn4*. Thus, one may suggest a commitment towards cellular proliferation rather than melanogenesis in *Opn4*^KO^ melanocytes.

Another important result is the interaction between increased *Mitf* and DNA repair genes. It has been reported that MITF controls UV-induced nucleotide excision repair genes, which ultimately leads to a faster transcription in response to DNA damage. Such events maintain an active transcriptional activity that is key for melanoma survival [70]. In fact, we found an increase of DNA repair-related genes such as *Atm* in *Opn4*^KO^ melanocytes, which may suggest that DNA repair in these cells could be faster and/or more efficient. In this line, we have previously shown that *Opn4*^KO^ melanocytes are less affected by low doses of UVA radiation [28], which results in reduced cellular growth in *Opn4*^WT^ melanocytes. Whether the lack of *Opn4* affected DNA repair capacity is a matter of further investigation.

As MITF is an important cell cycle regulator [12,13], increased gene expression may lead to downstream consequences. In fact, our data corroborate several studies in the literature: For instance, *Mitf* depletion from high or low *Mitf*-expressing melanoma cells results in cell cycle arrest [14,71]. BRAF has been shown to control MITF promoter through Octamer-Binding Transcription Factor 7 (BRN2). Remarkably, BRN2 expression is restricted to melanoblasts, and it is reactivated in melanoma cells during tumorigenesis by mutated BRAF, an event that does not take place in BRAF normal melanocytes [67]. In murine Melan-a melanocytes, ectopic expression of BRAF^V600E^ resulted in the activation ERK/MEK pathway, phorbol ester-independent growth, and tumorigenicity in mice, therefore, demonstrating that BRAF^V600E^ mutation is indeed oncogenic in normal melanocytes [72]. Another interaction of MITF with the cell is suggested by the suppression of melanoma colony formation by MITF downregulation, which can be rescued by CDK2 overexpression. Therefore, this study suggests that CDK2 is a downstream target of MITF [73]. In a physiological scenario, i.e., in melanocytes, MITF leads to increased cyclin-dependent kinase inhibitor 2A (p16^INK4A^) gene and protein levels, which results in retinoblastoma protein (pRb) hypophosphorylation. The consequence of such events is cell cycle arrest [74].

*Opn4* seems to interact with *Mitf*, as removal of *Opn4* resulted in increased *Mitf* expression, with important cell cycle alterations, which ultimately led to a faster proliferation. Such modifications may place *Opn4* as a putative tumor suppressor gene in melanoma. Within this line of thought, we previously reported that *Opn4*^KO^ melanocytes were more resistant to UVA-induced effects as daily low doses of UVA radiation reduced cellular growth only in *Opn4*^WT^ melanocytes [28]. Collectively, loss of OPN4 may result in a more resistant phenotype against the deleterious effects of UV radiation [28] and faster proliferation (these data), leading to an increased likelihood of mutation accumulation and cancer development.

Taken altogether, we have provided evidence that the lack of *Opn4* in murine melanocytes resulted in increased cellular growth and a faster cell cycle progression, which is associated with altered expression of gene/protein molecular clock components and increased *Mitf* expression, a master regulatory agent of melanocyte biology. A limitation of our study is a mechanistic view of how the lack of OPN4 affects *Mitf* expression and its downstream targets; however, in face of the phenotype and molecular clock gene alterations displayed by *Opn4*^KO^ melanocytes, we suggest a putative role of *Opn4* as a tumor suppressor gene in melanoma.

## Figures and Tables

**Table 1:** Primers, sequences, and access numbers.

Templates (Access #)	Primers and Probes (5′–3′)
***Atm*** **(NM_007499.3)**	For: AACCATGCTTGCTGTTGTCG Rev: AATCCAGCCAGAAAGCGTCA
***Atr*** **(NM_019864.1)**	For: CCTCAAACCGCTTTTTCGCA Rev: ATCCGGCCTTTTGTTGAGACT
***Ccna1*** **(NM_007628.3)**	For: GAAATTGCAGCTTGTCGGGA Rev: TGCCAGGACTTTGAGTAGCAG
***Chek1*** **(NM_007691.5)**	For: TGTGCATTTGGATTCCTGTGG Rev: CTATGGCCCGCTTCATGTCTA
***Ccnf*** **(NM_007634.4)**	For: TCCACGATGATGCACCCAAA Rev: TTTCTCGCTTCCGTTTGCTC
***Per1*** **(NM_0011065.3)**	For: AGCAGGTTCAGGCTAACCAGGAAT Rev: AGGTGTCCTGGTTTCGAAGTGTGT Probe: 5′-/6FAM/AGCCTTGTGCCATGGACATGTCTACT/BHQ_1/-3′
***Bmal1*** **(NM_001243048)**	For: AAGCTTCTGCACAATCCACAGCAC Rev: TGTCTGGCTCATTGTCTTCGTCCA Probe: 5′-/5HEX/AAAGCTGGCCACCCACGAAGATGGG/BHQ_1/-3′
***Clock*** **(NM_007715.6)**	For: CTCTGCTGCCTTTCCACTACAA Rev: TGCTGAGGCTGGTGTTGCT Probe: 5’-/5HEX/AGAGCACTTTCCCTCCTTCGCACCA/3BHQ_1/-3′
***Rev-Erbα*** **(NM_145434.4)**	For: AAGACATGACGACCCTGGAC Rev: CCATGCCATTCAGCTTGGTAAT
***Mitf*** **(NM_001113198.1)**	For: CCCAGGTATGAACACGCACT Rev: CTGTGGGGAAAATACACGCTG
***Xpa*** **(NM_011728.2)**	For: GGCGATATGAAGCTCTACCTAAA Rev: TTCCTGCCTCACTTCCTTTG
***Opn2*** **(NM_145383.1)**	For: TGCCACACTTGGAGGTGAAA Rev: ACCACGTAGCGCTCAATGG Probe: 5′-/6FAM/CGCCCTGTGGTCCCTGGTGG/BHQ_1/-3′
***Opn3*** **(NM_010098.3)**	For: GCTGCTTCTCTACTCCAAGTTCC Rev: TTCATAGGCCAGCACAGTGAG
***Rpl37a*** **(NM_009084.4)**	For: GCATGAAAACAGTGGCCGGT Rev: CAGGGTCACACAGTATGTCTCAAAA

## Data Availability

Data will be available upon reasonable request.
